# Type 1 diabetes complicated with uncontrollable adult cyclic vomiting syndrome: a case report

**DOI:** 10.1186/s40200-015-0206-6

**Published:** 2015-09-23

**Authors:** Kazuma Ogiso, Nobuyuki Koriyama, Ayako Akao, Mayumi Otsuji, Takahiko Goto, Natsuko Fujisaki, Machiko Minobe, Mayumi Kinowaki, Shigeru Matsuki

**Affiliations:** Department of Diabetes and Endocrine Medicine, National Hospital Organization Kagoshima Medical Center, 8-1 Shiroyama-cho, Kagoshima, 892-0853 Japan; Department of Diabetes and Nursing, National Hospital Organization Kagoshima Medical Center, 8-1 Shiroyama-cho, Kagoshima, 892-0853 Japan; Department of Diabetes and Clinical Psychologist, National Hospital Organization Kagoshima Medical Center, 8-1 Shiroyama-cho, Kagoshima, 892-0853 Japan; Department of Clinical Psychology, Graduate School of Clinical Psychology, Kagoshima University, 1-21-30 Korimoto, Kagoshima, 890-0065 Japan

**Keywords:** Type 1 diabetes mellitus, Cyclic vomiting syndrome (CVS), Continuous subcutaneous insulin infusion therapy (CSII), Hypnotherapy, Sandplay therapy

## Abstract

We herein describe the case of a 29-year-old woman with type 1 diabetes from 10 years of age who developed adult cyclic vomiting syndrome. Beginning at 25 years of age, she was frequently hospitalized for stress-induced vomiting. Her vomiting episodes developed acutely and remitted after severe vomiting of more than 30 times a day for a few days. The vomiting periods were accompanied by leukocytosis with a predominance of neutrophils, high blood pressure and fever. In addition, it was noted that her levels of both adrenocorticotropic hormone and antidiuretic hormone during the vomiting attacks increased and subsequently dramatically decreased immediately after symptom improvement; therefore, she was diagnosed with adult-type cyclic vomiting syndrome in accordance with the diagnostic criteria of Rome III, a system developed to classify functional gastrointestinal disorders. Though glycemic control had improved with continuous subcutaneous insulin infusion therapy, the vomiting frequency increased due to the failure of drug treatments and general psychotherapy to terminate the vomiting attacks, making discharge difficult and greatly interfering with everyday life. Eventually, hypnotherapy and miniature garden therapy were prescribed, which significantly reduced the vomiting frequency, making it possible to discharge her from inpatient medical care.

In the treatment of this patient with type 1 diabetes and adult-type cyclic vomiting syndrome, continuous subcutaneous insulin infusion therapy and comprehensive psychotherapy were effective.

## Background

In type 1 diabetes mellitus, it has been shown that the frequency of complications is reduced by the maintenance of good glycemic control [1]. In addition, it has been reported that patients with type 1 diabetes have more frequent complications with feeding behavior abnormalities and eating disorders [2, 3], as well as a significantly faster progression of complications [4]. It has especially been noted that complications of anorexia nervosa significantly increase mortality [5].

**Fig. 1:**
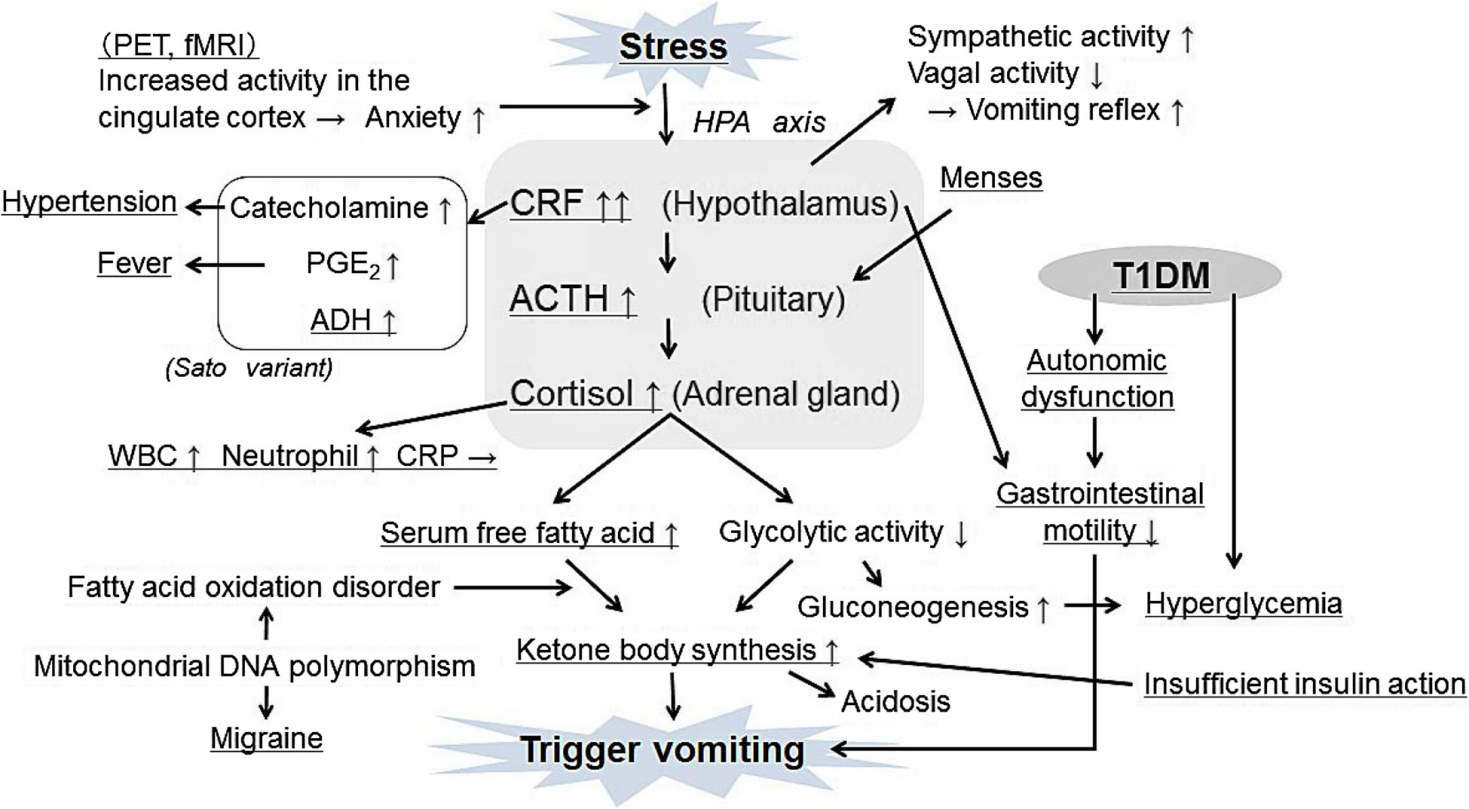
Pathologic hypothesis of disease. The secretion of ACTH and cortisol is increased by the oversecretion of CRF due to stress, while an increase in catecholamines, PGE2, and ADH is seen. Vomiting episodes are induced by promoting ketone body synthesis due to an increase in cortisol. The suppression of gastrointestinal motility by CRF is considered to be associated with vomiting. Increase in catecholamines and PGE2 may be related to hypertension and fever, respectively. If the family history of migraine is related to a fatty acid oxidation disorder caused by mitochondrial DNA polymorphism, it may promote the ketone body synthesis. Furthermore, it is considered that autonomic dysfunction and lack of insulin action associated with type 1 diabetes mellitus may facilitate vomiting attacks by the reduction of gastrointestinal motility and promotion of ketone body synthesis. In addition, menstruation and increased reactivity of the cingulate cortex to stress stimuli may be associated with excess secretion of CRF. The underlined text represents the findings observed in our case. PET, positron emission tomography; fMRI, functional MRI; HPA, hypothalamic-pituitary-adrenal axis; CRF, corticotropin releasing factor; ACTH, adrenocorticotropic hormone; PGE2, prostaglandin E2; ADH, antidiuretic hormone; CRP, C-reactive protein; T1DM, Type 1 diabetes mellitus

The periodic vomiting of psychogenic vomiting disease (cyclic vomiting syndrome or CVS) was reported in 1882 by Gee et al. [6], and is characterized by the periodicity of severe vomiting episodes, followed by remission, seen originally in 4-to 8-year-old children. Since the report of CVS in adults by Abell et al. for the first time in 1988 [7], the reported number of cases in adults has also gradually increased, and the concept of adult CVS (ACVS) has been established. In Japan, however, ACVS is not common at present, and is not often recognized. Currently, ACVS is defined as one of the functional gastrointestinal disorders by the Rome III criteria. Symptoms must persist for more than 6 months and must meet all of the following 3 criteria: (1) there is an episode of typical vomiting that remits in less than a week after acute onset, (2) there are more than 3 episodes of intermittent vomiting in 1 year and (3) there is an observed remission period between nausea or vomiting episodes during the last three months. In addition, as an auxiliary criterion, a personal or family history of migraine has been raised [8]. The origin of ACVS is thought to be a result of overreaction of the hypothalamic-pituitary-adrenal (HPA) axis at the time of psychological stress or infection. In particular, corticotropin releasing factor (CRF), which is secreted by the hypothalamus, is believed to be an important trigger as the vomiting phase begins. CRF, through stimulation of the hypersecretion of adrenocorticotropic hormone (ACTH) and cortisol, contributes to vomiting and promotes the synthesis of ketone bodies due to an increase in plasma fatty acids and the inhibition of glycolysis. Moreover, CRF also inhibits gastrointestinal motility via regulation of the autonomic nervous system (activation of the sympathetic nerve and suppression of the vagus nerve), and is thus believed to decrease mechanisms that inhibit vomiting [9–11]. In addition, it was revealed by Sato et al. in 1982 that ACTH, cortisol, catecholamines, antidiuretic hormone (ADH) and prostaglandin E2 (PGE2) in the peripheral bloodstream were increased by an excessive reaction of the HPA axis in one group of children. This is referred to as the Sato variant. That study particularly focused on patients with an increase in ACTH and ADH during the vomiting period, characterized by improvement and prompt remission, also known as ACTH-ADH hypersecretory syndrome [12–14]. Others [15] have reported that a family history of migraine is commonly recognized in patients with CVS. Another pathophysiologic possibility is mitochondrial dysfunction caused by mutations in mitochondrial DNA in which energy deficiency might trigger the onset of vomiting during increased energy demand at the time of infection or stress, as has been reported in pediatric patients [15–17]. Furthermore, because it has been reported that the reaction of regions of the brain associated with anxiety such as the cingulate cortex has changed [18], the response to stress in our patient might have been excessive. In addition to these factors, both the increased tendency toward synthesis of ketone bodies in type 1 diabetes with poor glycemic control and decreased gastrointestinal motility with autonomic neuropathy were also assumed to contribute to the vomiting attacks (Fig. 1).

Continuous subcutaneous insulin infusion therapy (CSII), since being used in many cases registered in the Diabetes Control and Complication Trial (DCCT) [1], has come to be widely recognized worldwide. CSII was demonstrated to have stronger hypoglycemic action in type 1 diabetes than multiple daily insulin injections (MDI). The insulin requirement was also decreased and was revealed to have a stabilizing effect on blood glucose levels [19]. The improvement with CSII was reported to be especially effective in patients with a hemoglobinA1c (HbA1c) value of more than 8 % [20]. However, in adolescent cases of type 1 diabetes, disease management with CSII is not as effective because glycemic control has also been shown to be unstable due to many other factors [20]. On the other hand, it has been demonstrated through meta-analysis that psychological interventions are associated with improvement in glycemic control [21]. In recent years, approaches that focus on psychological health have been important. Psychotherapies such as hypnotherapy and play therapy were included during traditional medical care. Hypnotherapy was developed based on the research of Erickson, a well-known psychiatrist and psychotherapist in the United States and has also been reported as being efficacious for Parkinson’s disease [22], migraine [23], anxiety and depression [24]. Sandplay therapy was developed by Swiss Jungian psychologist D.M. Kalff and has been defined as a psychotherapeutic method that enables patients to arrange miniature figures in a sandtray to create a “sandworld” corresponding to various dimensions of their social reality [25].

Here, we discuss a patient with type 1 diabetes complicated by ACVS who had repeated vomiting attacks that were especially difficult to control. We report that both CSII and comprehensive psychotherapy, including hypnotherapy and sandplay therapy, were effective.

## Case presentation

The patient was a 29-year-old woman who had been in good health until 1995, at which time she was diagnosed at 10 years of age with type 1 diabetes with a positive glutamate decarboxylase (GAD) antibody, and insulin treatment was started. Although her mother had a history of migraines, no history of diabetes in her family was observed.

Because she was bullied in elementary school due to diabetes, she developed overeating behaviors and her blood glucose levels became unstable; frequent hospitalizations were required and became pivotal to her daily life. Unfortunately, the patient continued to experience bullying even in junior high school, and her mother was often absent from home due to work; so the patient began overeating due to loneliness and anxiety. She was then allowed admission to a pediatric long-term care facility where she was also bullied by other patients. In addition, she was repeatedly hospitalized due to high plasma glucose levels. Ultimately, she entered a nursing-high school at the local hospital and graduated from junior high school. Following admission to the high school, to perform education through distance learning, she graduated at 20 years of age. During this period, she was hospitalized for a psychosomatic illness when she was 17 years old. She was transferred from the local hospital to another hospital’s pediatric unit. Furthermore, she began to display signs of retinopathy and overt proteinuria.

In September 2007, at 23 years old, the patient first visited our hospital through referral from the pediatrician at the previous hospital as she had become an adult and glycemic control was becoming increasingly difficult.

In December 2007, the patient, due to vomiting in response to stressful events, was first admitted to our hospital. On physical examination, her height was 158 cm, weight was 56.9 kg, BMI was 22.8 kg/m^2^, body temperature was 38.4 °C, blood pressure was 146/91 mmHg, and pulse was 117 bpm and regular. Her cardiopulmonary examination was normal. She had no abnormal abdominal findings. Her bilateral Achilles tendon reflexes were absent and bilateral lower extremity vibration sensation was also absent. On ophthalmologic examination, she had diabetic microangiopathies and growth arrest retinopathy. She demonstrated peripheral and autonomic neuropathy and had evidence of diabetic nephropathy of the third stage, i.e., overt proteinuria had been diagnosed, and her estimated glomerular filtration rate was 139.7 mL/min/1.73m^2^. She had no evidence of chronic thyroiditis (anti-thyroglobulin antibodies 11 IU/mL and anti-thyroid peroxidase antibodies 7 IU/mL; normal range: ≤ 28 IU/mL and ≤ 16 IU/mL, respectively).

**Fig. 2:**
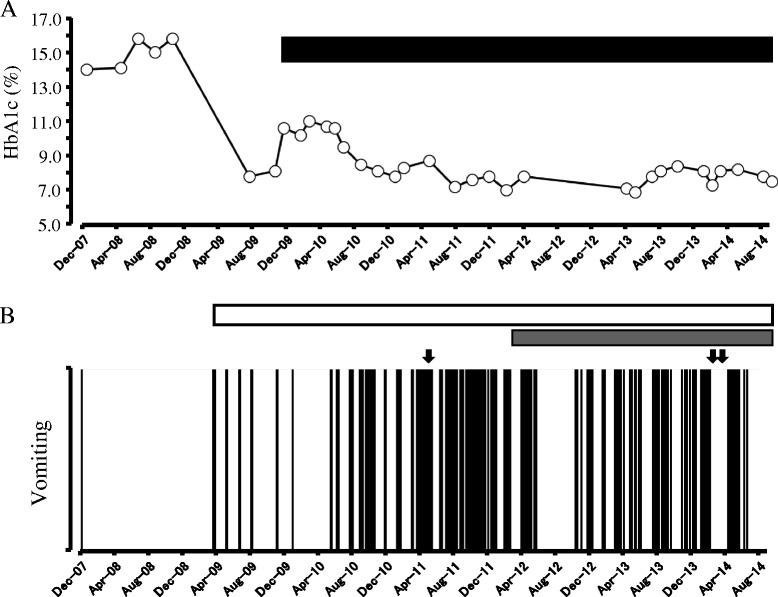
Transition of HbA1c (**a**) and vomiting episode frequency (**b**). **a**: Line graph linked by open circles shows the change in HbA1c; black rectangle shows the duration of continuous subcutaneous insulin infusion therapy. **b**: Histogram showing the episodes of vomiting attacks; white rectangle shows the duration of counseling, relaxation, cognitive behavioral therapy and psychological education by a clinical psychologist; gray rectangle shows the duration of hypnotherapy; and arrows indicate miniature garden therapy. During a total of 40 hospitalizations, nurses and doctors were committed to not only treating the patient medically but also providing emotional and social support

Due to financial difficulties, she was only able to afford outpatient visits once every 2 months. Her HbA1c at this time remained at 13 %. In April 2009, she was repeatedly hospitalized with dehydration and diabetic ketoacidosis (DKA) due to the interruption of insulin injections because of frequent vomiting that had appeared in response to stressful events. At that time, vomiting episodes occurred more than 30 times a day, and hematemesis was also observed. Nausea and increased salivation always persisted. Episodes were so severe that the patient was often found lying on the floor unresponsive and nearly unconscious. In addition, both fever and elevation of blood pressure were observed. For antiemetic therapy, intravenous administration of both diazepam and metoclopramide were ineffective, but intravenous administration of haloperidol was slightly effective. From around the 4^th^ or 5^th^ day after admission, vomiting gradually improved. During the period of vomiting, excessive secretion of both ACTH and ADH were observed, but they promptly improved during remission (Table 1). As a result of psychological intervention (behavioral therapy to include a combination of counseling and autogenic training, etc.) by a clinical psychologist, glycemic control showed temporary improvement (Fig. 2a). However, in December of the same year, we began CSII treatment during the vomiting phase because self-injection of insulin was interrupted and glycemic control had become more difficult with her HbA1c showing another upward trend (Fig. 2a). As a result, she no longer developed DKA during the periods of vomiting and was capable of maintaining her activities of daily living, while repeated cycles of vomiting remitted and glycemic control also showed a trend toward improvement (Fig. 2a, b).

**Table 1:** Laboratory data for the vomiting attack period and after improvement (April 2009)

		Vomiting period (Day 1)	Remission period (Day 6)	(Reference value)
ACTH	(pg/mL)	229 ↑	≤2.0↓	(7.2-63.3)
Cortisol	(μg/mL)	55.0 ↑	3.8 ↓	(4.0-18.3)
ADH	(pg/mL)	102 ↑	1.9	(0.3-3.5)
Serum osmolarity	(mOsm/kg⁠•H2O)	296 ↑	282	(276-292)
Acetoacetic acid	(μmol/L)	806 ↑	10	(≤55)
3-hydroxybutyric acid	(μmol/L)	2380 ↑	54	(≤85)
Total ketone bodies	(μmol/L)	3,186 ↑	64	(≤130)
WBC	(/μL)	14550^a^ ↑	7800	(3500-9000)
Neutrophil	(%)	86.2^a^ ↑	55.3	(42.0-74.0)
CRP	(mg/dL)	0.66^a^ ↑	<0.06	(<0.06)

^a^indicates the value on Day 3 of vomiting period

Subsequently, around March 2011, the patient’s vomiting episodes worsened and were more prolonged. She had a prolonged period of hospitalization, and her daily life again became difficult. Although her nutrition was managed by nasogastric tube feedings and central venous nutrition, her body weight decreased significantly to 38.0 kg with a body mass index (BMI) of 15.2 kg/m^2^.

In February 2012, we started hypnotherapy. The treatment was limited by poor language comprehension (Wechsler Adult Intelligence Scale-Third Edition [WEIS-III] score was 60 points), and a developmental disorder was inferred. Even so, the patient was able to be discharged because vomiting frequency was reduced by a combination of hypnotherapy and sandplay therapy (Fig. 2b). Today, her episodes of vomiting have not completely remitted, but it was possible to shift her to outpatient management, and her HbA1c has also been maintained at 7 %.

## Discussion

In our case, violent vomiting began more than 30 times a day without warning, and both nausea and vomiting disappeared within a week. Furthermore, there were more than 3 intermittent vomiting episodes per year, and there was a period of remission of nausea and vomiting between each episode. Migraine history was observed in the patient’s mother. From the above, she was considered to have met all of the diagnostic criteria. However, we also believed it was necessary to exclude celiac disease from the differential diagnosis, although the frequency is very low in Japan.

In this case, vomiting attacks triggered by stress appeared to evolve into a ketotic state with the observation of abnormally high levels of ACTH and ADH, which improved rapidly, along with recovery of vomiting (Table 1). Moreover, the elevation of leukocytes (predominantly neutrophils), hypertension and elevation of body temperature were observed during the vomiting period. We speculated that the increased neutrophil predominance of leukocytes without an increase in C-reactive protein (CRP) and the significant elevation of both blood pressure and fever reflected hypercortisolemia and the enhancement of catecholamine release as well as PGE2 production (catecholamines and PGE2 were not measured), respectively. Sato et al. reported the case of an 8-year-old girl with periodic attacks of vomiting. At the initiation of the attack, ACTH and ADH levels were prominently increased (610 pg/mL and about 82 pg/mL, respectively), followed by hypercortisolemia (51-80 μg/dL) [12]. Nakazato et al. described a 48-year-old woman with several attacks of vomiting. Her abnormal laboratory results included leukocytes (12,500/μL), CRP (0.54 mg/dL) and increased ACTH (196 pg/mL) [26]. Data from the cases shown in these reports were similar to those in our case (Table 1). In association with hormonal variation, vomiting attacks have often been found to overlap with menstruation. Shin et al. reported a case of cyclic vomiting syndrome in an adult patient characterized by stereotypical vomiting attacks occurring during every menstrual period [27]. Therefore, we considered it likely that this patient possessed elements of menstrual-related CVS.

A satisfactory therapeutic method for the treatment of ACVS has not yet been reported. So far, relaxation for stress prevention and medications such as tricyclic antidepressants with CRF inhibitory action, benzodiazepines with an anti-anxiety effect, 5-HT_3_ receptor antagonists with an antiemetic effect, antihistamines, D_2_ receptor antagonists and anti-migraine drugs (5-HT_10_ agonists) have been tried. However, many of them were ineffective in this case. In addition, very little antiemetic effect was observed with the administration of diazepam, metoclopramide and domperidone. Only haloperidol administration was relatively effective, but the mechanism was unknown. On the other hand, appropriate methods of coping with stress were considered to be important in the prevention of the onset of vomiting. Although we worked in conjunction with a clinical psychologist to improve the patient’s coping skills after remission of a vomiting attack, it was not possible to reduce the frequency of vomiting episodes. This is in contrast to the reported efficacy of hypnotherapy in the suppression of nausea and vomiting during cancer chemotherapy [28] as well as its remarkable antiemetic effect on hyperemesis gravidarum [29].

**Fig. 3:**
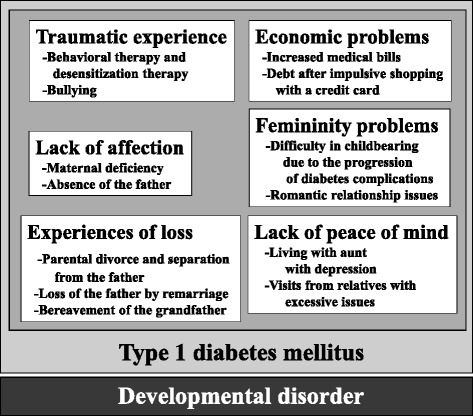
Origin of illness. The history of traumatic experiences, experiences of loss, lack of affection, economic problems, femininity problems and a lack of peace of mind, exist as an underlying basis in this patient’s developmental disorder in the background of complex type 1 diabetes

Kihlstrom et al. declared that hypnosis is a social interaction in which the subject (patient) responds to suggestions offered by the hypnotist (healer) for imaginative experiences involving alterations in conscious perception and memory and the voluntary control of action [30]. The healing principle has not been clarified yet; however, it is believed the hypnotic phenomenon engages the patient’s own problem-solving skills and develops the patient’s absolute trust in his or her proactive problem-solving ability. The understanding and use of the hypnotic phenomenon is an important communication tool between the patient and healer. From this standpoint, hypnotherapy can be expressed as non-verbal empowerment. It is believed that it is important to be sympathetic to the patients’ individual characteristic responses. In our patient, catalepsy, lacrimation, fear, an increase in body temperature and hypnotic responses indicating specific difficulties, such as colorless visions and feelings of floating than heaviness, were present. We presumed the relationship between CVS and harsh life events (traumatic experience, experience of loss, etc.) took place at a crucial time in this patient’s psychological development based on her developmental disorder and background of complex type 1 diabetes. At the same time, it was revealed that the origin of the illness could be related to underlying economic problems, lack of affection, etc. (Fig. 3), while the developmental disorder inferred by her WEIS-III score became a barrier to communication. However, when she was given suggestions under hypnotic induction and trance, it was possible for her to imagine a visual stimulus in a unique way to understand the various events. This was reinforced by sandplay therapy. With these interventions, there is a strong possibility that the effect of hypnotherapy raised her capacity for self-control of bodily sensations and emotions. As a result, the patient’s own emotional expression and self-insight were promoted with counseling and psychological education. Building a relationship with the patient and clarifying the roles of doctors and nurses continued, and those relationships appeared to function more effectively.

## Conclusions

We have reported a case of a patient with type 1 diabetes with ACVS. When the characteristic vomiting episodes are seen in patients with type 1 diabetes, it is necessary to take into account the possible overlay of ACVS. A combination of CSII, comprehensive psychotherapy including hypnotherapy and sandplay therapy and a commitment to the patient as a team, including a clinical psychologist, should be considered useful in attaining glycemic control and vomiting control for such patients.

## Consent

Written informed consent was obtained from the patient for publication of this case report and any accompanying images. A copy of the written consent is available for review by the Editor-in-Chief of this journal.

## References

[1] The Diabetes Control and Complications Trial Research Group (1993). The effect of intensive treatment of diabetes on the development and progression of long-term complications in insulin-dependent diabetes mellitus. N Engl J Med.

[2] Colton P, Olmsted M, Daneman D, Rydall A, Rodin G (2004). Disturbed eating behavior and eating disorders in preteen and early teenage girls with type 1 diabetes. Diabetes Care.

[3] Jones JM, Lawson ML, Daneman D, Olmsted MP, Rodin G (2000). Eating disorders in adolescent females with and without type 1 diabetes: cross sectional study. BMJ.

[4] Rydall AC, Rodin GM, Olmsted MP, Devenyi RG, Daneman D (1997). Disordered eating behavior and microvascular complications in young women with insulin-dependent diabetes mellitus. N Engl J Med.

[5] Nielsen S, Emborg C, Mølbak AG (2002). Mortality in concurrent type 1 diabetes and anorexia nervosa. Diabetes Care.

[6] Gee S (1882). On fitful or recurrent vomiting. St Bartholomew’s Hosp Rep.

[7] Abell TL, Kim CH, Malagelada JR (1988). Idiopathic cyclic nausea and vomiting--a disorder of gastrointestinal motility?. Mayo Clin Proc.

[8] Tack J, Talley NJ, Camilleri M, Holtmann G, Hu P, Malagelada JR (2006). Functional gastroduodenal disorders. Gastroenterology.

[9] Mayer EA, Naliboff BD, Craig AD (2006). Neuroimaging of the brain-gut axis: from basic understanding to treatment of functional GI disorders. Gastroenterology.

[10] Taché Y (2004). Corticotropin releasing factor receptor antagonists: potential future therapy in gastroenterology?. Gut.

[11] Taché Y (1999). Cyclic vomiting syndrome: the corticotropin-releasing-factor hypothesis. Dig Dis Sci.

[12] Sato T, Uchigata Y, Uwadana N, Kita K, Suzuki Y, Hayashi S (1982). A syndrome of periodic adrenocorticotropin and vasopressin discharge. J Clin Endocrinol Metab.

[13] Sato T, Igarashi N, Minami S, Okabe T, Hashimoto H, Hasui M (1988). Recurrent attacks of vomiting, hypertension and psychotic depression: a syndrome of periodic catecholamine and prostaglandin discharge. Acta Endocrinol.

[14] Wang Q, Ito M, Adams K, Li BU, Klopstock T, Maslim A (2004). Mitochondrial DNA control region sequence variation in migraine headache and cyclic vomiting syndrome. Am J Med Genet A.

[15] Sato T (1993). Prevalence of Syndrome of ACTH-ADH Discharge in Japan. Clin Prediatr Endocrinol.

[16] Boles RG, Williams JC (1999). Mitochondrial disease and cyclic vomiting syndrome. Dig Dis Sci.

[17] Boles RG, Chun N, Senadheera D, Wong LJ (1997). Cyclic vomiting syndrome and mitochondrial DNA mutations. Lancet.

[18] Namin F, Patel J, Lin Z, Dusing RW, Foran P, McCallum RW (2006). Recognizing abnormal patterns on PET brain images in adult patients with cyclic vomiting syndrome. Gastroenterology.

[19] Weissberg-Benchell J, Antisdel-Lomaglio J, Seshadri R (2003). Insulin pump therapy: a meta-analysis. Diabetes Care.

[20] Hirsch IB, Bode BW, Garg S, Lane WS, Sussman A, Hu P (2005). Insulin Aspart CSII/MDI Comparison Study Group. Continuous subcutaneous insulin infusion (CSII) of insulin aspart versus multiple daily injection of insulin aspart/insulin glargine in type 1 diabetic patients previously treated with CSII. Diabetes Care.

[21] Ismail K, Winkley K, Rabe-Hesketh S (2004). Systematic review and meta-analysis of randomized controlled trials of psychological interventions to improve glycaemic control in patients with type 2 diabetes. Lancet.

[22] Giovanni M, Giuseppe A, Franco V, Fiorenza B, Michela P, Francesco R (2000). Hypnosis in the treatment of anticipatory nausea and vomiting in patients receiving cancer chemotherapy. Oncology.

[23] Lindsey JW, John TR, Serdar HU (2012). Treatment of hyperemesis gravidarum. Rev Obstet Gynecol.

[24] Wain HJ, Amen D, Jabbari B (1990). The effects of hypnosis on a Parkinsonian tremor: case report with polygraph/EEG recordings. Am J Clin Hypn.

[25] Zhou D (2009). A review of sandplay therapy. Int J Psychol Stud.

[26] Nakazato Y, Tamura N, Shimazu K (2008). An adult case of cyclic vomiting syndrome successfully responding to valproic acid. J Neurol.

[27] Shin YK, Kwon JG, Kim KY, Park JB, Han SJ, Cheon JW, Kim EY, Lee TS, Park KS, Won KS (2010). A case of cyclic vomiting syndrome responding to gonadotropin-releasing hormone analogue. J Neurogastroenterol Motil.

[28] Ezra Y, Gotkine M, Goldman S, Adahan HM, Ben-Hur T (2012). Hypnotic relaxation vs amitriptyline for tension-type headache: let the patient choose. Headache.

[29] Willemsen R, Haentjens P, Roseeuw D, Vanderlinden J (2010). Hypnosis in refractory alopecia areata significantly improves depression, anxiety, and life quality but not hair regrowth. J Am Acad Dermatol.

[30] Kihlstrom JF, Nash MR, Barnier AJ (2008). The domain of hypnosis, revisited. Oxford handbook of hypnosis.

